# Why Is the Range of Timescale So Wide in Glass-Forming Liquid?

**DOI:** 10.3389/fchem.2020.579169

**Published:** 2020-09-29

**Authors:** Takeshi Egami, Chae Woo Ryu

**Affiliations:** ^1^Department of Materials Science and Engineering, Shull-Wollan Center – Joint Institute for Neutron Sciences, University of Tennessee, Knoxville, Knoxville, TN, United States; ^2^Department of Physics and Astronomy, University of Tennessee, Knoxville, Knoxville, TN, United States; ^3^Materials Sciences and Technology Division, Oak Ridge National Laboratory, Oak Ridge, TN, United States

**Keywords:** liquid, liquid dynamics, relaxation time, medium-range correlation, fragility

## Abstract

The viscosity and the relaxation time of a glass-forming liquid vary over 15 orders of magnitude before the liquid freezes into a glass. The rate of the change with temperature is characterized by liquid fragility. The mechanism of such a spectacular behavior and the origin of fragility have long been discussed, but it remains unresolved because of the difficulty of carrying out experiments and constructing theories that bridge over a wide timescale from atomic (ps) to bulk (minutes). Through the x-ray diffraction measurement and molecular dynamics simulation for metallic liquids we suggest that large changes in viscosity can be caused by relatively small changes in the structural coherence which characterizes the medium-range order. Here the structural coherence does not imply that of atomic-scale structure, but it relates to the coarse-grained density fluctuations represented by the peaks in the pair-distribution function (PDF) beyond the nearest neighbors. The coherence length is related to fragility and increases with decreasing temperature, and it diverges only at a negative temperature. This analysis is compared with several current theories which predict a phase transition near the glass transition temperature.

## Introduction

The viscosity of many liquids, such as water, is of the order of 10^−2^ poise (= 10^−3^ Pa.s). Its timescale, defined by the Maxwell relaxation time, τ_*M*_ = η/*G*_∞_, where η is viscosity and *G*_∞_ is the high-frequency shear modulus, is of the order of pico-second (ps). Upon cooling liquid viscosity rises rather quickly, if crystallization can be avoided for instance by fast cooling. At low enough temperatures τ_*M*_ becomes so long that the supercooled liquid behaves like a solid. This kinetically frozen liquid is a glass. The transition to the glassy state is defined by the value of η reaching 10^13^ poise (= 10^12^ Pa.s), when τ_*M*_ becomes of the order of 10^3^ s. Thus, the timescale of liquid dynamics changes by as much as 15 orders of magnitude over a moderate temperature range. Such a rapid change has direct implications on glass-forming ability and other properties of glass-forming liquids, as well as on applications. The origin of this large change has long been debated without wide agreement (Debenedetti and Stillinger, 2001; March and Tosi, 2002; Dyre, 2006; Lubchenko and Wolynes, 2007; Götze, 2009; Donth, 2010; Berthier and Biroli, 2011; Edigar and Harrowell, 2012; Parisi et al., 2020), and remains one of the glass mysteries.

Our recent research results suggest that the medium-range order (MRO) in liquid plays a crucial role in dynamics of metallic and other liquids (Ryu et al., 2019, 2020; Egami, 2020; Ryu and Egami, 2020). In this article we discuss these results and their wider implications in relation to other theories of liquids. In particular, we point out that our results do not suggest the divergence of viscosity just below the glass transition temperature, *T*_*g*_, as many other theories do, and provide a resolution to the Kauzmann paradox concerning entropy extrapolating to negative values at low temperatures (Kauzmann, 1948). Our results also challenge the idea that defect-like objects control atomic transport and deformation in liquid and glass and raise questions on some prevailing theories.

## Viscosity, Fragility and MRO

The temperature dependence of viscosity can be expressed in terms of the temperature dependent activation energy, *E*_*a*_(*T*), as

(1) $$\begin{array}{l} \eta \left(T\right)=\eta_{\infty }exp\left(\frac{E_{a}\left(T\right)}{k_{B}T}\right). \end{array}$$

Above the crossover temperature, *T*_*A*_, viscosity shows the Arrhenius behavior with a constant value of *E*_*a*_, and below *T*_*A*_ it becomes strongly super-Arrhenius (Angell, 1995; Kivelson et al., 1995), resulting in rapid increase in viscosity culminating to the glass transition. It has been shown by simulations (Iwashita et al., 2013) and by experiments (Iwashita et al., 2017; Shinohara et al., 2019; Ashcraft et al., 2020) that above *T*_*A*_ viscosity is determined by a bond cutting dynamics, and τ_*M*_ = τ_*LC*_, where τ_*LC*_ is the timescale for an atom to lose just one neighbor. Below *T*_*A*_, however, the τ_*M*_/τ_*LC*_ ratio increases rapidly with decreasing temperature, as liquid dynamics becomes more cooperative (Bellissard and Egami, 2018). This increase in cooperativity is the cause of the rapid increase in viscosity with decreasing temperature and eventual glass transition. The rate of increase in viscosity just above *T*_*g*_ is characterized by fragility,

(2) $$\begin{array}{l} {m=\frac{dlog\eta (T)}{d(T_{g}/T)}|}_{T_{g}}. \end{array}$$

A liquid with a large value of *m* is called fragile, whereas the one with a smaller value of *m* is called strong (Angell, 1995). The origin of the fragility is still in dispute (Angell, 1995; Novikov and Sokolov, 2004).

The structure of liquid and glass is usually described by the atomic pair-distribution function (PDF), *g*(*r*), which describes the distribution of distances between atoms by

(3) $$\begin{array}{l} g\left(r\right)=\frac{1}{4\pi r^{2}N\rho_{0}}\sum_{i,j}\langle \delta \left(r-|{\text{r}}_{\text{i}}-{\text{r}}_{\text{j}}|\right)\rangle , \end{array}$$

where ***r***_***i***_ is the position of the *i*-th atom, *i* = 1, …., *N*, δ(*r*) is the δ-function, ρ_0_ is the atomic number density, and <….> denotes thermal average. It is related to the structure function,

(4) $$\begin{array}{l} S\left(Q\right)=\frac{1}{4\pi Q^{2}N}\sum_{i,j}\text{exp}\left(i\text{Q}\cdot \left[{\text{r}}_{\text{i}}-{\text{r}}_{\text{j}}\right]\right), \end{array}$$

which can be determined by x-ray or neutron diffraction, through the Fourier-transformation,

(5) $$\begin{array}{l} g\left(r\right)=1+\frac{1}{2\pi^{2}\rho_{0}r}\int_{0}^{\infty }\left[S\left(Q\right)-1\right]\text{sin}\left(Qr\right)QdQ. \end{array}$$

According to Ornstein and Zernike (1914) the medium-range PDF beyond the first peak decays with *r* as

(6) $$\begin{array}{l} G\left(r\right)=4\pi r\rho_{0}\left[g\left(r\right)-1\right]=G_{0}\left(r\right)\text{exp}\left(-r/\xi_{s}\right), \end{array}$$

where *G*_0_(*r*) is the *G*(*r*) of the ideal glass, and ξ_s_ is the structural coherence length which characterizes the MRO. The ideal glass state defined by *G*_0_(*r*) has long-range density correlation without periodicity in the structure (Ryu et al., 2019). Because the medium-range PDF mostly accounts for the first peak of *S*(*Q*) (Cargill, 1975; Ryu et al., 2020), the height of the first peak, *S*(*Q*_1_)−1, where *Q*_1_ is the position of the first peak, is proportional to ξ_s_ (Ryu et al., 2019).

In Ryu et al. (2019) *G*(*r*), thus ξ_s_, was measured for Pd_42.5_Ni_7.5_Cu_30_P_20_ liquid by high-energy x-ray diffraction using electrostatic levitation, from 420 to 1,100 K through the glass transition (573 K). Just above *T*_*g*_ *E*_*a*_(*T*) was found to be directly related to ξ_s_ by,

(7) $$\begin{array}{l} E_{a}\left(T\right)=E_{0}{\left(\frac{\xi_{s}\left(T\right)}{a}\right)}^{3}, \end{array}$$

where *a* is the average neighbor distance (Ryu et al., 2019) and *E*_0_ is a scaling parameter. Because

(8) $$\begin{array}{l} n_{c}\left(T\right)=\rho_{0}{\left(\xi_{s}\left(T\right)\right)}^{3} \end{array}$$

is the number of atoms in the coherence volume, ${\left(\xi_{s}\right)}^{3}$, it is indicative of the degree of atomic cooperativity of local dynamics in liquid. In other words, *E*_*a*_(*T*) is proportional to the number of atoms involved in the activation process for viscous flow;

(9) $$\begin{array}{l} E_{a}\left(T\right)=n_{c}\left(T\right)E_{B},\;E_{B}=\frac{E_{0}}{\rho_{0}a^{3}}. \end{array}$$

*E*_*B*_ represents the bond energy per atom, which is of the order of a fraction of eV and is significantly larger than *k*_*B*_*T*_*g*_, whereas *n*_*c*_ is relatively small even at *T*_*g*_, typically below ten. The ratio of *E*_*B*_/*k*_*B*_*T*_*g*_ being larger than unity allows small changes in cooperativity *n*_*c*_(*T*) resulting in large changes in *E*_*a*_, and the rapid increase in viscosity below *T*_*A*_. Moreover, for various liquids examined by experiments as well as by simulations it was found that *n*_*c*_ at *T*_*g*_ is directly linked to fragility by,

(10) $$\begin{array}{l} n_{c}\left(T_{g}\right)=\frac{m}{m_{0}}, \end{array}$$

where *m*_0_ = 8.7 overall, 10.7 for metallic liquids, 7.4 for organic liquids, and 7.3 for network liquids (Ryu and Egami, 2020). Thus, fragility is related to the cooperativity of liquid dynamics and also to the “ideality” of the liquid structure. The liquid ideality is defined by the shape of the first peak of *S*(*Q*) being close to Lorentzian, as implied by the Ornstein-Zernike form, Equation (6), and by long ξ_s_ (Ryu et al., 2020). The Equation (9) appears similar to that of the classical Adam-Gibbs theory (Adam and Gibbs, 1965) in which the critical size of the cooperatively rearranging region, z^*^, determines viscosity. However, the coherence volume defined here refers to the correlation in bulk liquid in equilibrium, whereas the cooperatively rearranging region is a transient defective object. This point will be discussed below.

## Temperature Dependence of MRO and Viscosity

In Ryu et al. (2019) we studied the temperature dependence of the structure for various liquids by experiments and simulations, and showed that the height of the first peak of the structure function, *S*(*Q*), and the coherence length follow the Curie-Weiss law,

(11) $$\begin{array}{l} \xi_{s}\left(T\right)=\frac{C}{T-T_{IG}}, \end{array}$$

where *T*_*IG*_ is the ideal glass temperature which is negative. The origin of this behavior was briefly discussed in terms of the atomic-level stresses Egami (2011) in Ryu et al. (2019), and will be explained elsewhere (Egami and Ryu, 2020). Then at *T*_*g*_,

(12) $${\frac{d}{d\left(T_{g}/T\right)}\left(\frac{\xi_{s}\left(T\right)}{\xi_{s}\left(T_{g}\right)}\right)|}_{T_{g}}=\frac{1}{1-T_{IG}/T_{g}}=m_{c},$$

**Figure 1:**
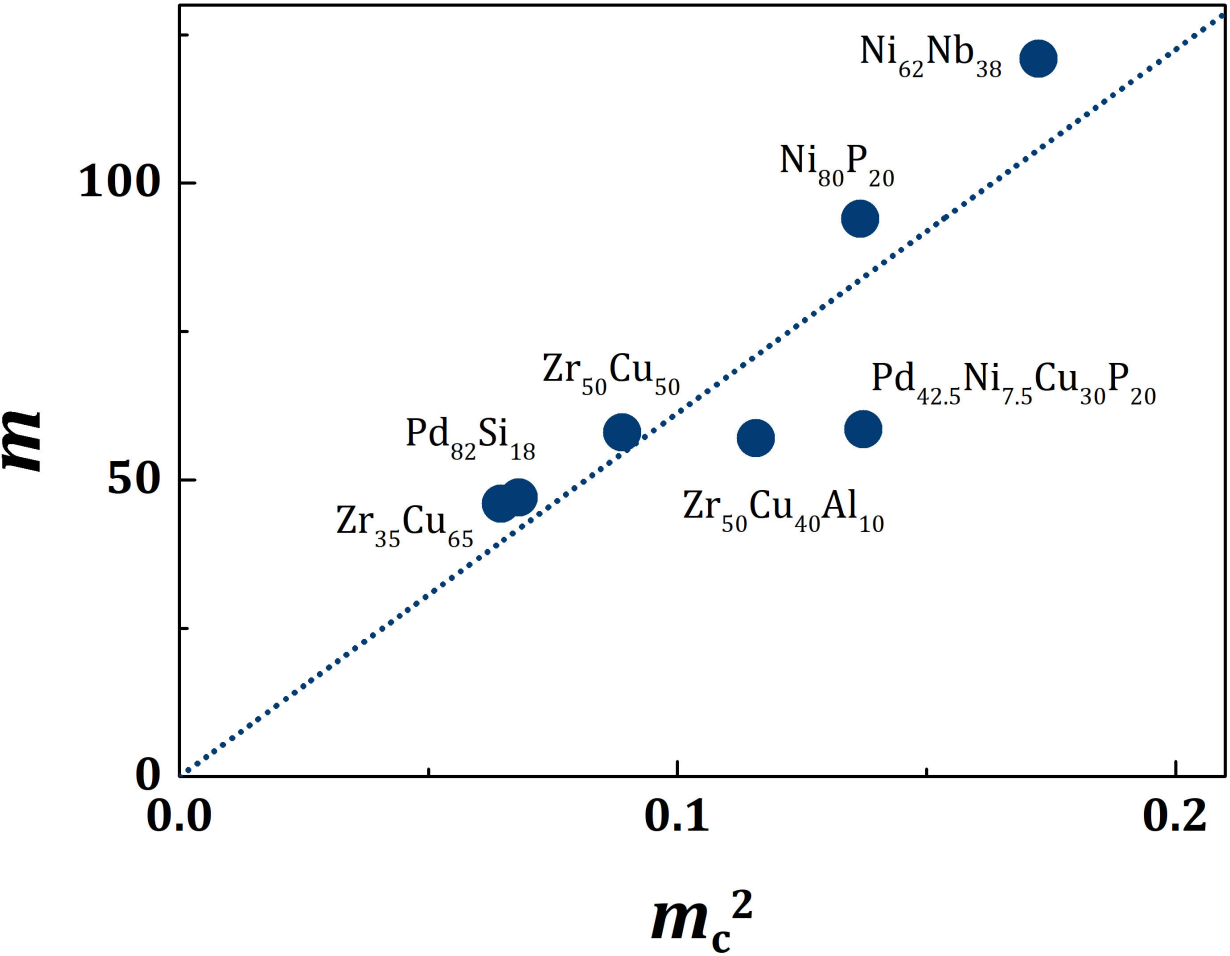
The plot of *m*_*c*_^2^ vs. *m* for various metallic liquids. The dotted line is for *m*_1_ = 613 in Equation (13).

We found that,

(13) $$\begin{array}{l} m_{c}^{2}=\frac{m}{m_{1}}. \end{array}$$

with *m*_1_ = 613, as shown in Figure 1 for various metallic alloy liquids. Therefore,

(14) $$\begin{array}{l} \frac{T_{IG}}{T_{g}}=1-{\left(\frac{m_{1}}{m}\right)}^{1/2}. \end{array}$$

Because

(15) $$\begin{array}{l} \rho_{0}=\frac{f_{p}}{V_{a}}=\frac{6f_{p}}{\pi a^{3}}, \end{array}$$

where *V*_*a*_ is the atomic volume and *f*_*p*_ is the atomic packing fraction,

(16) $$\begin{array}{l} \frac{a}{\xi_{s}\left(T\right)}=\frac{{\left(m\right)}^{\frac{1}{6}}}{{\left(m_{1}\right)}^{1/2}{\left(\frac{\pi }{{6m}_{0}f_{p}}\right)}^{1/3}}\left(\frac{T}{T_{g}}-\frac{T_{IG}}{T_{g}}\right), \end{array}$$

(17) $$\begin{array}{l} \frac{C}{aT_{g}}=\frac{{\left(m_{1}\right)}^{1/2}{\left(\frac{\pi }{{6m}_{0}f_{p}}\right)}^{1/3}}{m^{1/6}}. \end{array}$$

**Figure 2:**
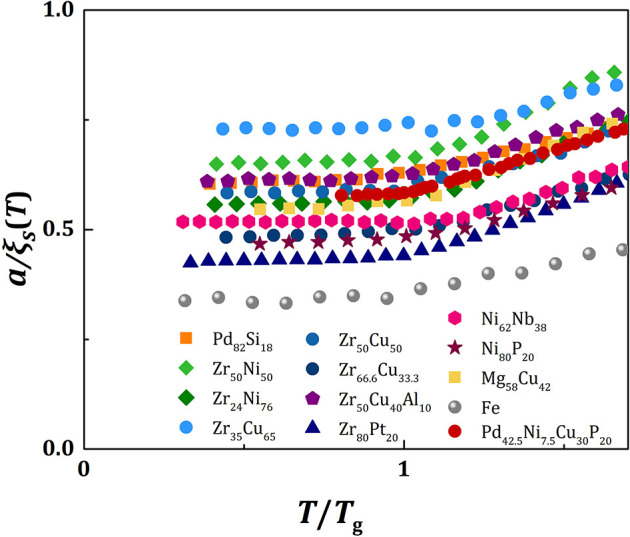
The plots of *a*/ξ_*s*_(*T*) against *T/T*_*g*_ for various metallic liquids.

**Figure 3:**
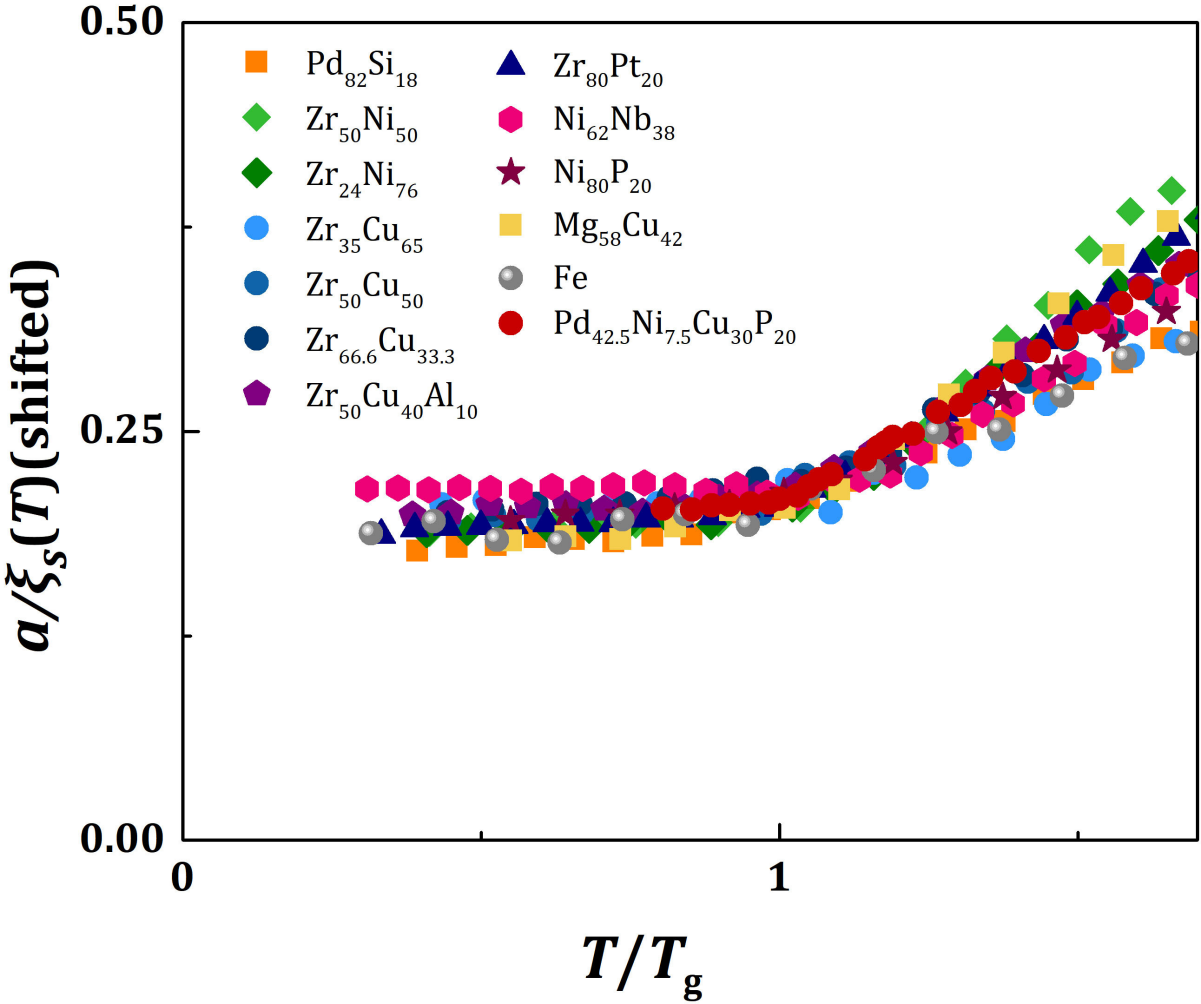
The plots of *a*/ξ_*s*_(*T*) against *T/T*_*g*_ for various metallic liquids with vertical shifts to form a near universal curve.

**Figure 4:**
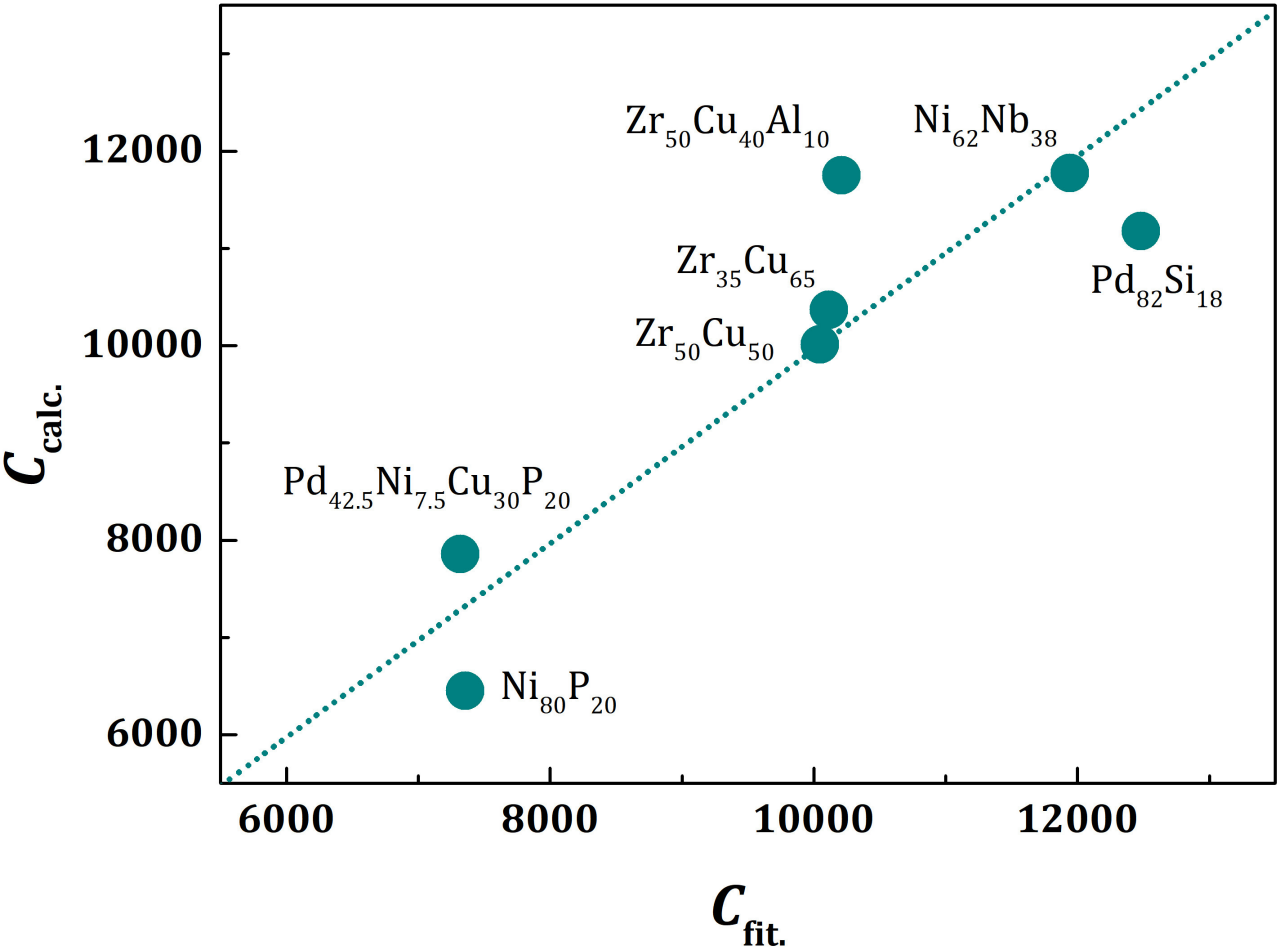
The value of *C* given by Equation (17), *C*_*calc*_, plotted against the value of *C* fit by Equation (11), *C*_*fit*_, for various liquids. The dotted line is a linear fit, which is virtually identical to the line for *C*_*calc*_ = *C*_*fit*_.

Because the value of *f*_*p*_ is similar for all metallic glasses (~0.7), the plots of *a*/ξ_*s*_(*T*) against *T/T*_*g*_ should be similar, except for vertical shifts, as shown in Figure 2. With vertical shifts they collapse to a near universal curve up, except for weak dependence above *T*_*g*_ on *m* (Figure 3). The value of *C* calculated by Equation (17), *C*_*calc*_, is compared to the value of *C* obtained by fit with Equation (11), *C*_*fit*_, for various liquids in Figure 4, showing good agreement. This near universality must be the reason for the success of the Kivelson scaling (Kivelson et al., 1995).

From Equations (1, 7, 17) we have

(18) $$\begin{array}{l} \eta \left(T\right)=\eta_{\infty }exp\left(\frac{E_{0}}{k_{B}T}{\left(\frac{T_{1}}{T-T_{IG}}\right)}^{3}\right), \end{array}$$

where

(19) $$\begin{array}{l} T_{1}=\frac{{\left(m_{1}\right)}^{1/2}{\left(\frac{\pi }{{6m}_{0}f_{p}}\right)}^{1/3}}{m^{1/6}}T_{g}. \end{array}$$

**Figure 5:**
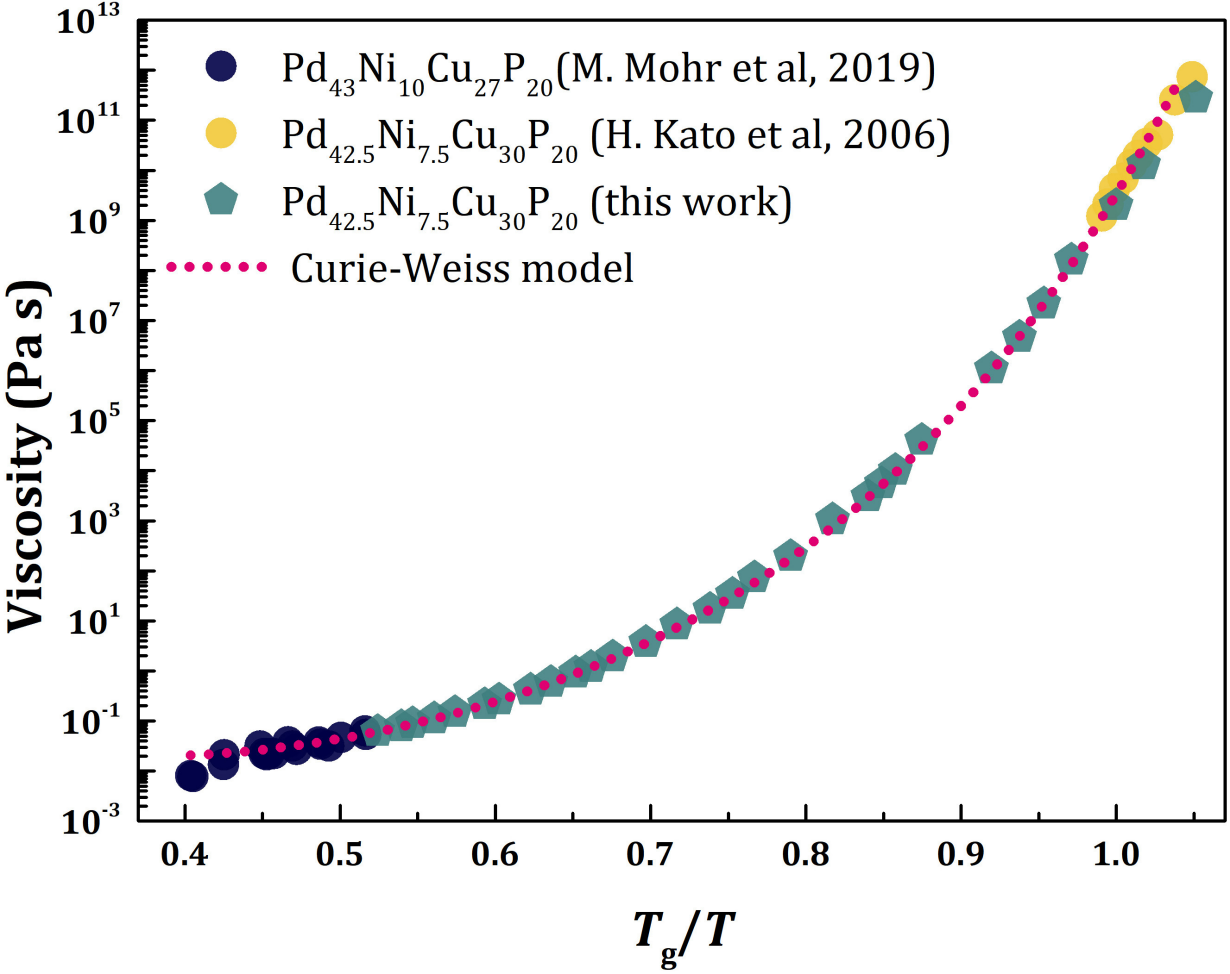
The temperature dependence of viscosity of PdNiCuP liquid: Experimentally determined viscosity data of PdNiCuP liquid (Kato et al., 2006; Mohr et al., 2019) compared to those calculated. The pentagonal symbol denotes the viscosity calculated with the ξ_*s*_(*T*) determined from the PDF measured by x-ray diffraction (Ryu et al., 2019) using Equation (20), whereas the dashed line was calculated using the Curie-Weiss law, Equation (11).

Thus, the viscosity just above *T*_*g*_ can be described in terms of *E*_0_ and *T*_*IG*_. At temperatures above *T*_*A*_ the value of *E*_*a*_ becomes constant (= *E*_∞_) even though ξ_s_ keeps decreasing. The crossover is a purely dynamic phenomenon (Iwashita et al., 2013), and the MRO is irrelevant to dynamics above *T*_*A*_. With a reasonable crossover, for instance,

(20) $$\begin{array}{l} E_{a}\left(T\right)=E_{\infty }{\left(\frac{\xi_{s}\left(T\right)}{b}\right)}^{d\left(T\right)}, \end{array}$$

where *d*(*T*) = 3 for *T* < *T*_*g*_, $d\left(T\right)=3\left(\frac{T_{g}}{T}-\frac{T_{g}}{T_{A}}\right)/\left(1-\frac{T_{g}}{T_{A}}\right)$ for *T*_*g*_ < *T* < *T*_*A*_, and *d* = 0 for *T* > *T*_*A*_, a realistic temperature dependence of viscosity can be reproduced as shown in Figure 5. Here we compare the experimentally determined viscosity data of PdCuNiP liquid (Kato et al., 2006; Mohr et al., 2019) with those calculated with the Equation (20). The pentagonal symbol denotes the viscosity calculated with the ξ_*s*_(*T*) determined from the PDF measured by x-ray diffraction (Ryu et al., 2019), whereas the dashed line is calculated using the Curie-Weiss law, Equation (11). We assumed *T*_*A*_*/T*_*g*_ = 2.0 (Blodgett et al., 2015), used the high-temperature data (Mohr et al., 2019) to determine the values of *E*_∞_ (= 0.77 eV) and η_∞_ (= 1.82 × 10^−5^ Pa.s), and the low-temperature data (Kato et al., 2006) to determine the value of *b* (= 3.72 Å).

## Comparison With Other Theories and Models

### Absence of Divergence

The divergence of viscosity was first predicted by the Vogel-Fulcher-Tamman (VFT) model (Vogel, 1921; Fulcher, 1925; Tammann and Hesse, 1926),

(21) $$\begin{array}{l} \eta \left(T\right)=\eta_{0}exp\left(\frac{B}{T-T_{0}}\right). \end{array}$$

Models based upon structural coherence, such as the icosahedral correlation models (Steinhardt et al., 1981; Tomida and Egami, 1995; Tanaka et al., 2010), predict the divergence of structural coherence, thus the divergence of viscosity, below *T*_*g*_ in the vicinity of the Kauzmann temperature, *T*_K_ (Kauzmann, 1948). The mode-coupling theory (Götze, 2009) predicts the divergence at a temperature, *T*_*c*_, which is even higher than *T*_*g*_, and only defect hopping provides mobility below *T*_*c*_ (Biroli et al., 2006). For a long time, it has been difficult to measure the viscosity of simple liquids above *T*_*g*_ because of crystallization. In the absence of serious questioning many of the current theories still assume the divergence of viscosity in the vicinity of *T*_K_.

However, a more recent measurement of viscosity using liquid levitator (Blodgett et al., 2015) suggests that the VFT model actually shows poor fit to the data. It is likely that the prediction of the viscosity divergence is based upon poor extrapolation of viscosity to infinity. In fact, many other models do not predict divergence at *T* > 0 (Cohen and Grest, 1979; Nussinov, 2004; Demetriou et al., 2006; Elmatad et al., 2009; Mauro et al., 2009). According to Equation (7) viscosity diverges when the coherence length ξ_s_(*T*) diverges. For metallic glasses the value of ξ_*s*_(*T*_*g*_)/*a* ranges from 1 to 2.7, with the average around 1.8. Therefore, the structure is quite far from ideal even at *T*_*g*_. The temperature at which the ideal state is achieved in extrapolation, *T*_*IG*_, is negative. Thus, viscosity never diverges at *T* > 0 and entropy does not become negative, resolving the Kauzmann paradox (Kauzmann, 1948).

### Nature of Structural Order

Many theories attribute the origin of increased viscosity to development of some structural order which is frustrated and cannot achieve long-range ordering. The most prominent example of such order is the icosahedral order (Sadoc, 1981; Steinhardt et al., 1981). The idea is that because the icosahedral order is incompatible with periodicity it never grows into long-range order (Nelson, 1983; Sethna, 1983). However, such structural orders depend on chemical composition and local chemistry (Gaskell, 1979). Also, this is just a sophisticated version of the nano-crystalline theory which Frank (1952) tried to disprove by suggesting the possible presence of local icosahedral configuration. Note that liquid is stabilized by configurational entropy: The development of local order of a particular atomic configuration will reduce the entropy and destabilize liquid.

On the other hand, we postulate the ideal liquid/glass state by extrapolating the coherence length ξ_s_ to infinity (Ryu et al., 2019). This state has very diverse local structures, with widely varying local configurations. For instance, the population of the icosahedral local structure is merely 0.7%. The order parameter, ξ_s_, does not describe the structural order, but the MRO of local density fluctuation. Higher-order peaks of the PDF at large distances include many interatomic distances within the peak. The width of the high-order PDF peaks is about 0.1 nm, and this defines the spatial resolution of the structure in the ideal state. Thus, the MRO describes coarse-grained density fluctuations, and not the atomic-level structural correlations, because the spatial resolution needs to be better at least by an order of magnitude to specify the atomic structure. In our view the local icosahedral ordering which occurs in single element liquid is not indicative of glass formation, but it is likely be that of nano-scale crystallization or quasicrystal formation. A single-element metallic liquid is a very poor glass-former and easily crystallizes. For a single-component liquid the second peak of *S*(*Q*), which is more sensitive to crystallinity, diverges at a positive temperature below *T*_*g*_ (Ryu et al., 2019). This suggests that the divergence of the local order just below *T*_*g*_ implies nano-scale crystallization.

### Idea of Defects

In crystalline solids atomic transport occurs only through the motion of lattice defects, such as vacancies and interstitial defects. Because the structure of liquid and glass is strongly disordered and appears to be full of defect-like structures, it was only natural to assume that more defective parts of the structure allow easier atomic transport. This led to many ideas of defects in liquid and glass, including free-volume (Cohen and Turnbull, 1959), cooperatively rearranging region (Adam and Gibbs, 1965), shear-transformation-zone (Argon, 1979), and others including ours (Egami et al., 1980). However, the results above suggest that the bulk properties, the MRO, control atomic transport, not those of defects. Would the concept of defect be still relevant in elucidating the atomic transport? Our answer is that the concept of defect defined by the specific static structure is not applicable to liquid and glass. We have to consider the “structure” as a dynamic entity.

It has been recognized for a long time that the definition of defect in amorphous system is arbitrary, in the absence of the reference structure. Various attempts have been made to define the defects, by studying the nature of the static structure, including the approaches using machine-learning (Cubuk et al., 2015; Bapst et al., 2020). However, it became apparent recently that what matters is the dynamics, not the static structure before deformation. In crystalline solids the defect retains its structural identity even after motion, because of the translational symmetry of the host lattice. In other words, defects are topologically protected by the lattice. In liquid and glass, however, the topology of atomic connectivity is open, and defects are not topologically protected. The atomic configuration before the motion of a defect is very different from that after the motion. In the picture of the potential energy landscape (PEL), the system moves from one valley to the other through a saddle-point. It was fond that, at the saddle-point, the potential energy of the system is high enough for the system to melt locally for a very short time (~1 ps) (Ding et al., 2020). Consequently, the system loses the memory of prior thermal history (Fan et al., 2017). The saddle-point is known to be a generator of chaos (Mason and Piiroinen, 2012; Párraga et al., 2018). The simple, usually hand-written, schematic picture of the PEL gives an impression that the pathway from one valley to the next is pre-determined. However, in reality, the kinetic momenta of atoms, which vary rapidly in time, give rise to large uncertainty in the directions toward which the system evolves. The major virtue of the PEL concept is that by removing the kinetic energy the underlying PEL is clearly exposed. However, to describe the dynamics of the system we need to add back the kinetic energy which introduces uncertainty, particularly at the saddle-point.

The local melting at the saddle-point decouples the pathway from a valley of the PEL up to the saddle-point and the pathway down to another valley. Therefore, what determines the nature of the saddle-point, thus the system dynamics, is not the initial state in the prior valley but the nature of the molten state which reflects the bulk property, such as the MRO. The propensity to start the activation process depends on the energy of the initial state, which can be described in terms of the fictive, or effective, temperature in the glassy state (Langer, 2004; Fan et al., 2017). However, once the process of activation over the PEL saddle point starts it does not matter where it started initially. The dynamics of the system at the saddle point is totally controlled by the bulk properties, the coherence volume, to be specific.

### Mode-Coupling Theory

The mode-coupling theory (MCT) is one of the most widely used theories of liquid dynamics. It describes the dynamics in terms of continuum hydrodynamic variables, such as density and current auto-correlation functions. It is based on the Boltzmann-type equation of motion initially developed for colloids. In the equation of motion, the dynamics at time *t* is coupled to the dynamics at a prior time *t'* through the memory function which represents the frictional force. The input to the theory is the snapshot structure function, *S*(*Q*), particularly its first peak. Because the height of the first peak of *S*(*Q*) is proportional to ξ_s_ (Ryu et al., 2019), the MCT focuses on the MRO, similarly to our approach. The dynamic correlations are determined by the equation of motion, and the feedback through the memory function determines dynamics, leading to the glass transition.

In colloids, particles are in touch with solvent, which is in the hydrodynamic steady state, so that the use of the frictional term in the Boltzmann equation is justified. However, in atomic liquids atoms interact each other directly via the potential force, so the application of the MCT becomes more contorted. Viscosity is given in terms of the stress autocorrelation via the Green-Kubo equation. In the MCT this retention of stress correlation is expressed as the memory function which gives rise to the frictional force. Therefore, the feedback from the memory function can produce a runaway leading to the divergence of correlation time.

In our approach the dynamics is governed by discrete local atomic activation processes. The probability of activation is controlled by the activation energy which is directly related to the structural coherence length ξ_s_. The ξ_s_ is an equilibrium property, which depends only on temperature and the elastic constants through the atomic-level stresses, without the feedback loop through the memory function. In colloids local dynamics is closely coupled to local density fluctuations, because density plays the role of temperature in the hard-sphere system. Hard jamming at the critical density leads to divergence of viscosity. In atomic liquids, however, hard jamming never occurs, because atoms are compressible and thermal activation is always possible. Even though the MCT explains the glass transition of colloidal systems, its applicability to atomic liquids should be examined more carefully.

### Infinite Dimension Models

The spin-glass theories of Edwards and Anderson (1975) and Sherrington and Kirkpatrick (1975) used the replica method (Aharony, 1975; Emery, 1975) and established the presence of the spin-glass ground state, at least in the infinite dimensions. In many spin-glasses spins interact through the long-range RKKY interaction. The large number of interacting neighboring spins justifies the use of the mean-field approximation. The replica method was applied later to the glass problem (Mézard and Parisi, 2000).

In spin-glasses randomness is quenched, because the spin Hamiltonian does not change with temperature. In contrast in real liquids and glasses the Hamiltonian varies with time and temperature. The number of atoms involved in action, *n*_*c*_, is small. Therefore, a similar mean-field approximation is more difficult to justify, and atomic discreteness becomes central to the dynamics. For instance, at *T*_*A*_, ξ_*s*_(*T*_*g*_)/*a* ≈ 1, so in Equation (7) *E*_0_ represents the bond energy and *n*_*c*_, ~ 2. The dynamics is totally local, and the action of cutting a bond determines viscosity and diffusivity. Even at *T*_*g*_, *n*_*c*_ ranges from 2 to 12, whereas the ideal state, where ξ_*s*_(*T*_*g*_)/*a* → ∞, is achieved only at a negative temperature. Thus, the liquid above *T*_*g*_ is very far from the ideal state. The infinite dimension theories may be justified in the ideal state, but they may not be appropriate for the real glass and liquid which are far removed from the ideal state. The glass theories based on exact solutions in infinite dimensions (Parisi et al., 2020) are beautiful, but the success of its application to real liquids and glasses needs to be proven.

## Conclusions

The study of the structural medium-range-order (MRO) in metallic liquids, represented by the coherence length, ξ_s_, through diffraction experiment and simulation shows that the MRO is intimately related to local dynamics and viscosity. Namely the activation energy of viscosity is directly related to the number of atoms involved in local atomic rearrangement for structural excitation, *n*_*c*_(*T*), which is proportional to (ξ_s_)^3^. The magnitude of *n*_*c*_(*T*) is relatively small, 2 ~ 12 even at *T*_*g*_, so that the discrete nature of the atomic structure, represented by the topology of atomic connectivity network, is crucial. Conversely, it means that the unit energy for activation per atomic bond, *E*_*B*_, is relatively large. Therefore, a small increase in *n*_*c*_(*T*) would result in large increase in the activation energy and viscosity. In our view this must be the reason why the timescale of liquid dynamics changes so rapidly over a moderate temperature range. At the same time, the system is quite far from the point of viscosity divergence which occurs when *n*_*c*_(*T*) diverges to infinity. Actually, we predict *n*_*c*_(*T*) to diverge at a negative temperature, by extrapolation with the Curie-Weiss law. Therefore, the Kauzmann catastrophe never occurs at *T* > 0. Even though the results presented here focus on metallic liquids, the same approach was successful in elucidating the ideality and fragility of network and some organic liquids (Ryu and Egami, 2020; Ryu et al., 2020), suggesting that this approach may be applicable beyond metallic liquids. This view is at odds with some of the theories and ideas. This conflict will be resolved by further theoretical and experimental advances in the future.

## Data Availability Statement

All datasets generated for this study are included in the article.

## Author Contributions

This work was conceived and authored by TE with assistance by CR. The data in this work were generated and analyzed by CR. Both authors contributed to the article and approved the submitted version.

## Conflict of Interest

The authors declare that the research was conducted in the absence of any commercial or financial relationships that could be construed as a potential conflict of interest.
